# Can Rhomboid Intercostal Block Be an Alternative to Paravertebral Block in Video-Assisted Thoracoscopic Surgery? A Randomized Prospective Study

**DOI:** 10.3390/diagnostics14192129

**Published:** 2024-09-25

**Authors:** Mete Manici, Belitsu Salgın, Muhammet Selman Söğüt, Serhan Tanju, Şükrü Dilege, Yavuz Gürkan, Hesham Elsharkawy

**Affiliations:** 1 Department of Anesthesiology and Reanimation, Koç University Hospital, Davutpaşa Caddesi No:4, Topkapı, Istanbul 34010, Turkey; mmanici@kuh.ku.edu.tr (M.M.); ssogut@kuh.ku.edu.tr (M.S.S.); yavuzgurkanmd@gmail.com (Y.G.); 2 Department of Thoracic Surgery, Koç University Hospital, Davutpaşa Caddesi No:4, Topkapı, Istanbul 34010, Turkey; stanju@kuh.ku.edu.tr (S.T.); sdilege@ku.edu.tr (Ş.D.); 3 Anesthesiology, Pain and Healing Center, MetroHealth, Case Western University, Cleveland, OH 44106, USA; h_sharkus@yahoo.com; 4 Outcomes Research Consortium, Cleveland, OH 44109, USA

**Keywords:** interfascial blocks, rhomboid intercostal block, VATS, postoperative analgesia

## Abstract

Background/Objectives: Rhomboid intercostal block (RIB) is a new interfascial plane block. RIB is a simple and clinically effective technique. Paravertebral block (PVB) is offered as a first-line regional anesthesia technique for thoracoscopic surgeries. In this study, we aim to compare the analgesic efficacy of RIB to PVB in video-assisted thoracoscopic surgeries (VATSs). Methods: This is a prospective randomized study with 84 patients aged 18–75 and ASA I–III, undergoing VATS for primary lung cancer. The study was approved by an ethical committee and registered under clinicaltrials.org. With informed consent, patients were randomized to receive ultrasound-guided RIB or PVB at T5-level with 20 mL of %0.25 bupivacaine preoperatively. Surgeries were performed under general anesthesia. Postoperatively, patient-controlled IV fentanyl analgesia was prescribed, delivering 10 μg boluses upon request with 10 min of a lock-out period. Patients received paracetamol 1 g IV three times a day and tramadol 50 mg IV for breakthrough pain. The postoperative Numeric Rating Scale (NRS) for pain, total opioid consumption, and rescue analgesic requirements were recorded postoperatively at 1, 3, 6, 12, and 24 h. Results: There were no significant differences in 24 h total opioid consumption between the RIB and PVB groups [PVB: 48.5 (39.5–55) mcg; RIB: 48.6 (40.2–65) mcg; *p* = 0.258], nor in rescue analgesic requirements [PVB: seven patients (20%); RIB: seven patients (17.1%); *p* = 0.570]. NRS pain scores were also similar between the groups, with no significant difference in overall pain control efficacy (*p* = 0.833). Conclusions: RIB is comparable to PVB in analgesic efficacy for VATS and can be considered as an alternative analgesic modality.

## 1. Introduction

Video-assisted thoracoscopic surgery (VATS) has emerged as the less invasive and preferred method for pulmonary oncologic surgery. This minimally invasive technique has revolutionized thoracic surgery by significantly reducing the trauma associated with traditional open surgeries. The advantages of VATS include decreased postoperative pain levels and lower stress hormone responses, which collectively contribute to a more favorable recovery profile for patients. These benefits extend to better-preserved pulmonary function in the early postoperative phase, which is crucial for early mobilization and enhanced recovery, reducing postoperative complications such as deep vein thrombosis and pneumonia, thus improving overall patient outcomes and shortening hospital stays [1]. Despite these advantages, patients undergoing VATS still experience moderate pain during the first postoperative hours. Thoracic epidural anesthesia (TEA) has long been considered the gold standard for managing postoperative pain in thoracic surgeries due to its efficacy. However, TEA is not without its drawbacks; it carries the risk of potential complications such as hypotension, urinary retention, and motor blockade, which can significantly impact patient recovery and overall satisfaction.

Current guidelines have moved away from recommending TEA due to its higher complication and side effect rates [2]. The PROSPECT guidelines for VATS postoperative pain management now recommend less invasive regional anesthetic techniques as the first line of treatment. Specifically, paravertebral block (PVB) and erector spinae plane block (ESP) are recommended for their effectiveness in managing postoperative pain with a reduced risk of complications. PVB has demonstrated non-inferior analgesic efficacy compared to TEA and has gained acceptance as the new gold standard for VATS [3]. The efficacy of PVB is comparable to that of TEA in terms of pain relief, but with the added benefit of better hemodynamic stability. This includes a lower incidence of hypotension, reduced need for perioperative fluid resuscitation, and decreased use of vasopressors such as phenylephrine [4].

Although rhomboid intercostal block (RIB) is a relatively new technique, first described by Elsharkawy et al. in 2016, it has quickly garnered attention for its efficacy in reducing pain and perioperative opioid consumption across a variety of clinical scenarios [5]. Cadaveric studies have demonstrated that RIB provides extensive coverage of the thoracic dermatomes, specifically from T3 to T9, covering both the anterior and posterior aspects of the hemithorax. This wide craniocaudal spread ensures comprehensive pain relief across a significant portion of the thoracic region, making it a versatile option for various surgical procedures [6]. RIB consists of the injection of a local anesthetic deep into the rhomboid muscles, into the tissue plane between the ribs, the external intercostal muscles, and the rhomboid muscles along the medial border of the scapula [7]. This placement allows for the effective blockade of the intercostal nerves, thereby providing significant analgesia.

Rhomboid intercostal block has demonstrated effectiveness in thoracoscopic surgeries [8,9,10,11,12]. Although there are a limited number of reports, available evidence suggests that RIB can provide significant pain relief and reduce opioid requirements in patients undergoing minimally invasive thoracic procedures. In this study, the objective was to compare the analgesic efficacy of RIB (intervention) to that of PVB (comparison) in patients undergoing oncologic thoracoscopic pulmonary surgery (population) by evaluating its effect on total opioid consumption in the postoperative first 24 h (primary outcome), pain scores, and rescue analgesic consumption (secondary outcomes).

## 2. Materials and Methods

### 2.1. Ethics Statement

This study is designed as a randomized prospective clinical study. Ethical committee approval was obtained from Koç University Committee on Human Research, under the number 2021.066.IRB1.022. The study was registered on ClinicalTrials.gov, under the identifier NCT05061667, with the first submission date of 29 September 2021. The enrolment period was between January 2022 and December 2022. Written informed consent was obtained from all recruited patients.

### 2.2. Patient Selection and Randomization

ASA I–III patients, aged 18–75 years old, undergoing primary oncologic video-assisted thoracoscopic wedge resection or lobectomy under general anesthesia were selected for this study. Exclusion criteria included pregnancy, bleeding diathesis, chronic pain medication use, renal insufficiency, infection at the block site, patient refusal to participate in the trial, or known allergy to used medications.

The participants were randomized into two groups using the software system of randomizer.org (http://www.randomizer.org, accessed on 20 December 2021). Group I received a PVB, while Group II received an RIB. The randomization was basic, non-blocked, and non-categorized.

Both the patients and the outcome assessors were blinded to the interventions. After randomization, patients were unaware of whether they had received the rhomboid intercostal block or the paravertebral block. The anesthesiologists performing the blocks could not be blinded due to the nature of the procedures, but they were not involved in postoperative pain management or data collection. All postoperative analgesic consumption and pain scores were recorded by pain management nurses, who were blinded to the intervention group assignments.

All surgeries were performed by the same surgical team, using a 2-port approach, with one port designated for the camera and the other for access. All patients were primary lung cancer patients. Postoperative chest tube drainage was inserted in all patients at the fifth intercostal space and removed on postoperative day 2.

### 2.3. Nerve Blocks

All nerve blocks were performed by a single, non-blinded, experienced anesthetist in the operating room, immediately after patients were monitored using the standard ASA guidelines, before the induction of anesthesia. Due to the procedural requirements, the anesthetist could not be blinded to the group allocation. However, the surgeons were blinded to patient allocation to prevent any bias in surgical technique or postoperative care.

#### 2.3.1. Paravertebral Block

**Figure 1:**
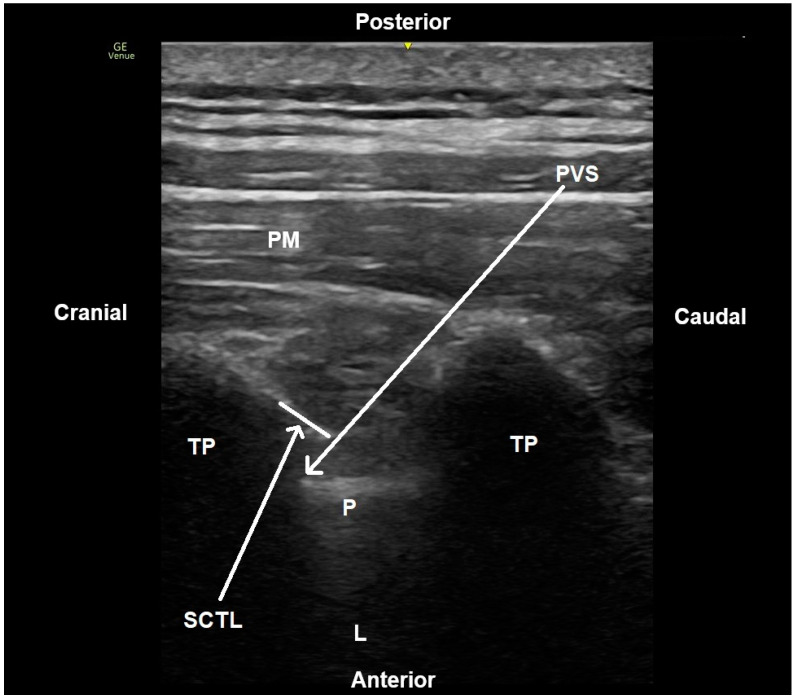
Ultrasound image of paravertebral block process. PM: paraspinal muscle, TP: transverse process, SCTL: superior costotransverse ligament, PVS: paravertebral space (arrow representing the needle trajectory), P: pleura, L: lung.

The paravertebral block was performed with the patient in a sitting position to facilitate optimal access to the thoracic spine and ensure the accurate visualization of anatomical landmarks. Continuous monitoring was established, and a high-frequency linear ultrasound probe (8–18 MHz) from the GE Logiq S7 system (General Electric Healthcare, Chicago, IL, USA) was positioned at the T5-T6 vertebral level. The probe was then moved laterally by approximately 2–3 cm to visualize key structures, including the pleura, transverse processes, and the paravertebral space (Figure 1). A 50 mm, 22G regional block needle (BBraun, Kassel, Germany) was introduced in-plane under real-time ultrasound guidance, directed toward the paravertebral space. Once the transverse process was contacted, the needle was “walked off” the process, advancing 1–2 cm deeper into the paravertebral space. Proper placement was confirmed by the downward displacement of the pleura upon the injection of saline or local anesthetic. Following negative aspiration to avoid vascular puncture, 20 mL of 0.25% bupivacaine (Marcaine^®^ 0.5%, AstraZeneca PLC, London, UK) was injected. An ultrasound was used to confirm the craniocaudal spread of the local anesthetic, and pleural displacement was observed, which indicated the successful delivery of the block.

#### 2.3.2. Rhomboid Intercostal Block

**Figure 2:**
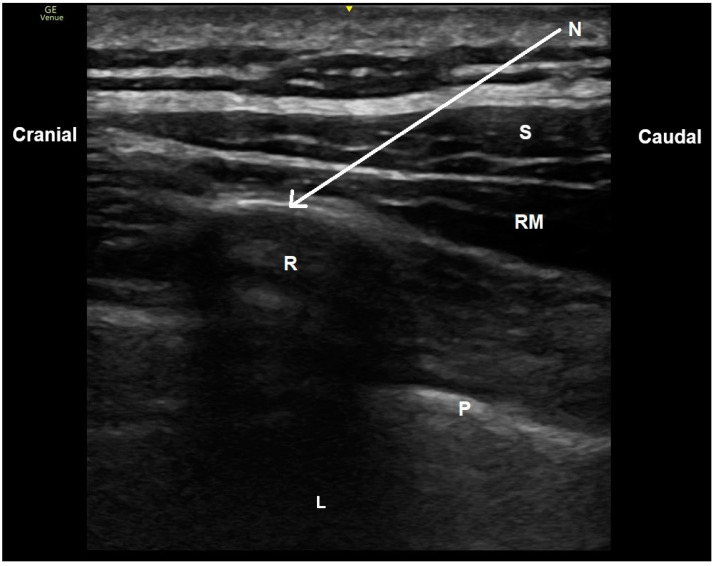
Ultrasound image of rhomboid intercostal block process. S: serratus anterior muscle, RM: rhomboid muscle, R: rib, L: lung, N (arrow): needle trajectory.

The rhomboid intercostal block was performed with the patient in the lateral decubitus position to allow optimal access to the rhomboid and intercostal muscles [5]. Following the method described by Elsharkawy et al., the procedure began with the placement of a high-frequency linear ultrasound probe (8–18 MHz) from the GE Logiq S7 system (General Electric Healthcare, Little Chalfont, UK) horizontally along the medial border of the scapula at the T5–T6 vertebral level. The anatomical landmarks, including the rhomboid major muscle, intercostal muscles, and the pleura, were identified. A 50 mm, 22G needle (BBraun, Kassel, Germany) was introduced in-plane, targeting the fascial plane between the rhomboid major and intercostal muscles. After ensuring negative aspiration, 20 mL of 0.25% bupivacaine was slowly injected under ultrasound guidance. The injection was monitored in real time to confirm the correct placement of the needle and the spread of the anesthetic, visualizing the separation of the muscle layers (Figure 2).

### 2.4. Perioperative Management

Following nerve blocks, general anesthesia was induced for all groups with propofol 2–3 mg/kg, fentanyl 2 µg/kg, and rocuronium 0.6 mg/kg. Anesthesia maintenance was achieved with 1.0 MAC desflurane and 0.02–0.2 µg/kg/min remifentanil infusion. The depth of anesthesia was monitored with a bispectral index range of 40–60 using BIS monitoring. Ondansetron 4 mg was used for PONV prophylaxis. Before emergence from anesthesia, intravenous paracetamol 1 g and dexketoprofen 50 mg was administered for postoperative analgesia. Postoperatively, patient-controlled IV analgesia with fentanyl was prescribed, delivering 10 μg boluses with 10 min of lock-out period and no background infusion. As part of multimodal analgesia, all patients received paracetamol 1 g IV three times a day until discharge. In cases where patients reported NRS scores exceeding 3, tramadol 50 mg IV was administered to ensure adequate pain relief.

### 2.5. Data Collection and Statistical Analysis

The primary outcome of the study was the total difference in fentanyl consumption at 24 h postoperatively. Secondary outcomes were the pain scores, opioid consumptions, and rescue analgesic consumptions at 1, 3, 6, 12, and 24 h. Whether or not a patient required rescue analgesia was recorded at each postoperative survey hour, and the need was tracked using a binary system where 0 indicated no need and 1 indicated the need for rescue analgesia. Secondary endpoints were the overall patient satisfaction and incidence of nausea and vomiting. The study hypothesized that RIB has comparable analgesic efficacy to PVB for VATS postoperative pain.

To evaluate the non-inferiority of the RIB compared to PVB, the study established a non-inferiority margin of d = 5, with a type I error (α) set at 0.05 and a type II error (β) at 0.80. Based on these assumptions, the sample size was calculated to require 41 patients in each group, providing a robust statistical basis for comparison.

Data analysis was conducted using SPSS software (IBM, Armonk, NY, USA), version 20.0. The distribution of the data was initially assessed using the Shapiro–Wilk test, which determined whether the results followed a normal distribution. The homogeneity of variances was examined with the Levene test. Depending on the distribution and homogeneity of the data, either the *t*-test or the Mann–Whitney U test was applied to compare the means of the two groups and determine if there were any significant differences.

For the statistical comparison of time-series data, including opioid consumption, NRS scores, and rescue analgesic consumption, R (version 4.2.3 (15 March 2023 ucrt)) and the nparLD package (version 2.2 (7 August 2022)) were used [13]. This package is designed for non-parametric longitudinal data analysis and was used to conduct rank-based tests for time and group effects. Specifically, the Wald-type and ANOVA-type tests were applied to assess overall differences between the two groups, the effect of time, and the interaction between time and group. Relative treatment effects (RTEs) were calculated to quantify differences in rank means between groups over time. The significance level for all statistical tests was set at *p* < 0.05.

## 3. Results

### 3.1. Patient Demographics

**Figure 3:**
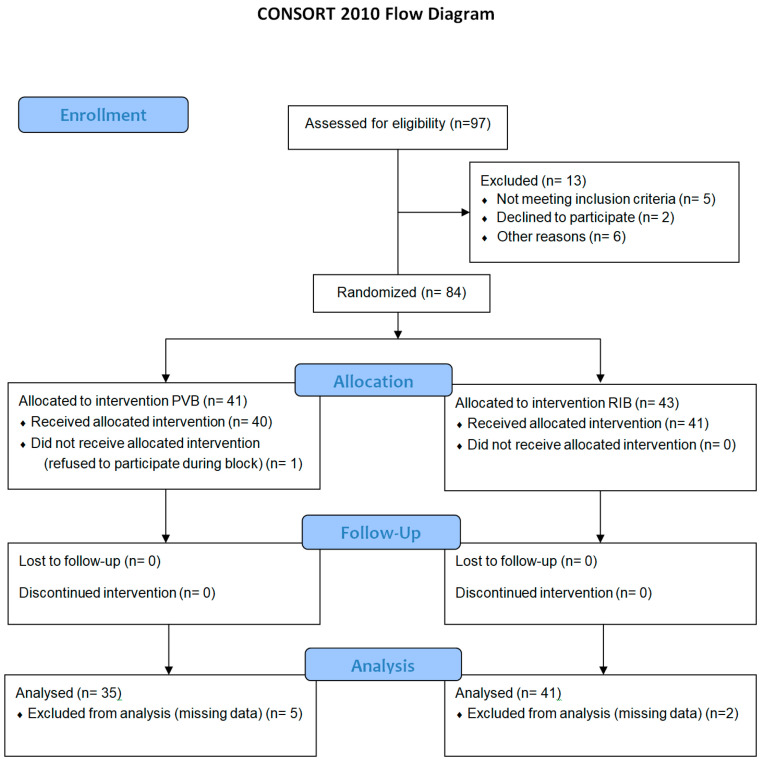
Consort 2010 flow diagram.

A total of 97 patients were screened for inclusion in this study. Of those screened, 84patients were enrolled and randomly allocated into two groups. The data of 76 patients were statistically analyzed. Figure 3 shows a CONSORT diagram of patient enrolment.

The demographic and clinical characteristics of the patients in the PVB and RIB groups are summarized in Table 1. The median age of patients differed between the groups, with the PVB group having a higher median age of 65.8 years (IQR 61–72.5) compared to 61 years (IQR 53–67.3) in the RIB group. This difference was statistically significant, with a *p*-value of 0.002.

### 3.2. Opioid Consumption

In terms of total opioid consumption, there were no significant differences between the RIB and PVB groups over the 24 h postoperative period. The RIB group required a median of 48.6 mcg (IQR 40.2–65) of fentanyl, while the PVB group had a median consumption of 48.5 mcg (IQR 39.5–55). The difference in fentanyl consumption between the two groups was not statistically significant, with a *p*-value of 0.258.

**Figure 4:**
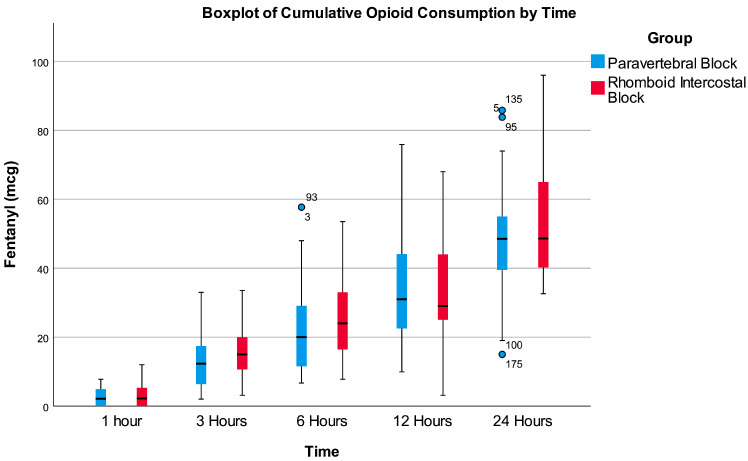
Boxplot of cumulative opioid consumption by time.

The time-series analysis of opioid consumption in the 1st, 3rd, 6th, 12th, and 24th hours using the Wald and ANOVA tests for the non-parametric analysis of longitudinal data indicated no significant difference between the two groups in overall opioid consumption (*p* = 0.235). Opioid consumption increased significantly over time in both groups (Wald test: *p* < 0.001; ANOVA test: *p* < 0.001). The interaction between block type and time was not significant (*p* = 0.555), meaning that the rate of increase in opioid consumption over time did not differ significantly between the two groups (Figure 4).

### 3.3. Secondary Outcomes

**Figure 5:**
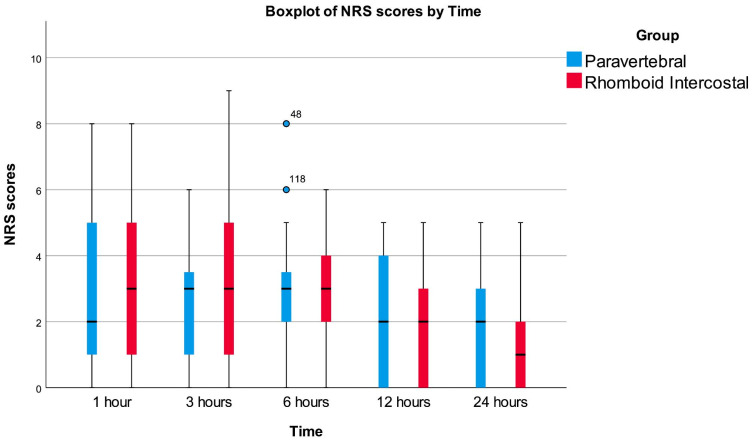
Boxplot of NRS scores by time.

Postoperative pain was assessed using the Numeric Rating Scale at five time points: 1 h, 3 h, 6 h, 12 h, and 24 h after surgery. The Wald and ANOVA tests for the non-parametric analysis of longitudinal data showed no significant difference between the two block groups overall (*p* = 0.833). The effect of time on pain scores was highly significant (Wald test: *p* < 0.001; ANOVA test: *p* < 0.001), suggesting a significant reduction in NRS scores over time in both groups. The interaction between block type and time was not statistically significant (*p* = 0.329), indicating that the trend of decreasing NRS scores was similar across both block groups (Figure 5).

In total, seven patients (20%) in the PVB group and seven patients (17.1%) in the RIB group required rescue analgesia. There was no statistically significant difference in overall rescue analgesic consumption between the two groups in the time-series analysis (Wald test: *p* = 0.571; ANOVA test: *p* = 0.572). However, the effect of time on rescue analgesic usage was highly significant (Wald test: *p* < 0.001; ANOVA test: *p* < 0.001), indicating that rescue analgesic consumption varied significantly over time. The interaction between group and time was not statistically significant (*p* = 0.663), suggesting similar trends in rescue analgesic use across both groups over time.

The relative treatment effect for rescue analgesic usage in the PVB group (RTE = 0.506) was slightly higher than in the RIB group (RTE = 0.493), but this difference was not statistically significant.

The incidence of postoperative nausea was higher in the RIB group (11 patients, 26.8%) compared to the PVB group (5 patients, 14.3%). However, the difference was not statistically significant (χ^2^(1, *n* = 75) = 1.79, *p* = 0.181).

## 4. Discussion

The primary outcome of this study focused on total opioid consumption within the first 24 h after surgery. No significant differences were observed between the rhomboid intercostal block (RIB) and paravertebral block (PVB) groups. The median fentanyl consumption was 48.6 mcg (IQR 40.2–65) in the RIB group and 48.5 mcg (IQR 39.5–55) in the PVB group, with no statistically significant difference (*p* = 0.258). These findings align with results from Wang et al., who reported no significant difference in analgesic efficacy between RIB and PVB for postoperative pain management following video-assisted thoracoscopic surgery (VATS) [14]. Time-series analysis of opioid consumption also showed no significant interaction between block type and time (*p* = 0.555), indicating that both blocks resulted in similar opioid consumption patterns over time.

In terms of pain control, both groups demonstrated significant reductions in NRS pain scores over time, with no significant difference between the RIB and PVB groups (*p* = 0.833). These results are consistent with other studies, such as the trial by Wang et al., which also reported similar postoperative pain control outcomes between RIB and PVB [14]. Another trial by Wang et al. confirmed that RIB was non-inferior to PVB, showing comparable pain scores and opioid usage [15].

Rescue analgesic consumption also showed no significant difference between the groups, with 20% of patients in the PVB group and 17.1% in the RIB group requiring additional pain relief. Although the time effect was significant for rescue analgesic use, the interaction between group and time was not significant, suggesting that both block types resulted in similar rescue analgesic use patterns. These findings highlight that both RIB and PVB provide comparable pain relief during the early postoperative period.

Comparing the efficacy of RIB to other regional anesthesia techniques, studies have shown that RIB is on par with traditional methods like PVB. Zhao et al. conducted a network meta-analysis that demonstrated the effectiveness of PVB, intercostal nerve block (ICNB), and thoracic epidural anesthesia (TEA) in reducing chronic post-surgical pain (CPSP) following VATS, while the serratus anterior plane block (SAPB) was less effective [16]. Although long-term outcomes such as CPSP were not assessed in this study, the comparable short-term analgesia provided by RIB and PVB suggests that both techniques are effective for managing postoperative pain.

Further research has also explored the potential benefits of combining RIB with other blocks, such as the subserratus plane block (RISS). Deng et al. demonstrated that the combination of RIB and RISS provided superior postoperative pain relief and reduced opioid consumption compared to RIB alone [17]. Similarly, Kozanhan et al. and Zhang et al. reported that the RISS block was associated with reduced pain scores, decreased analgesic consumption, and better-preserved lung function when compared to intravenous analgesia [18,19]. These findings suggest that combining RIB with other blocks could enhance its analgesic efficacy.

In contrast, the erector spinae plane block (ESPB) has been shown to be less effective compared to PVB. A study by Filho et al. found that ESPB was not non-inferior to PVB in lung surgeries, with ESPB patients experiencing higher postoperative pain scores and requiring more opioids [20]. This suggests that while ESPB is a viable option, it may not provide the same level of postoperative analgesia as PVB or RIB in thoracic surgery.

This study has several limitations that should be acknowledged. First, the anesthetist performing the blocks was not blinded to group allocations, which may have introduced potential bias. Second, the total intraoperative remifentanil use was not recorded, which could have provided a more comprehensive evaluation of the blocks’ effectiveness in managing intraoperative pain and reducing opioid consumption. Additionally, the absence of a control group prevents comparison between RIB, PVB, and a standard intravenous patient-controlled analgesia protocol.

Another limitation of our study is the exclusion of two patients from the rhomboid intercostal block group and five patients from the paravertebral block group due to missing or incomplete data. While this reduced the sample size slightly, it is unlikely to have influenced the overall findings, as the statistical results were clear and not close to the significance thresholds.

Another limitation is the age difference between the groups, as the PVB group was significantly older. Age-related factors may have influenced pain perception, opioid consumption, and reporting accuracy. The use of the Numeric Rating Scale (NRS) for pain assessment, while practical, may have been challenging for some older patients, particularly those with cognitive impairments such as dementia or delirium. Although Charlson’s Comorbidity Index (CCI) was not used, it could provide additional insight into the influence of comorbidities on pain perception.

Lastly, the small sample size may affect the reliability and generalizability of the findings. A larger sample would allow for more robust data and stronger conclusions. While non-parametric methods were used due to non-normal distribution, a normally distributed dataset could have enabled parametric testing for more generalizable statistical interpretations.

## 5. Conclusions

In conclusion, these findings suggest that RIB provides postoperative analgesia comparable to PVB, with no significant differences in opioid consumption, pain scores, or rescue analgesic use within the first 24 h following surgery. Both blocks are effective in managing postoperative pain, and combining RIB with other techniques, such as the subserratus plane block, may offer enhanced pain relief and improved recovery. Overall, RIB presents as a valuable alternative to traditional techniques like PVB in postoperative pain management for thoracic surgery. Further studies are needed to evaluate the effectiveness of RIB in larger patient groups and multiple centers.

## Figures and Tables

**Table 1:** Patient demographics by group.

	PVB (*n* = 35)	RIB (*n* = 41)	*p*
Gender *n* (%)			0.577 ^1^
Male	20 (57.1)	26 (63.4)	
Female	15 (42.9)	15 (36.6)	
Age Med (IQR)	68 (61–72.5)	61 (53–67.3)	0.002 ^2^
ASA Score *n* (%)			0.319 ^3^
I	1 (2.9)	5 (12.2)	
II	25 (71.4)	27 (65.9)	
III	9 (25.7)	9 (22)	
Type of Surgery			0.558 ^1^
Lobectomy	19 (54.3)	19 (47.5)	
Wedge	16 (45.7)	21 (52.5)	

^1^ Yates’ continuity correction, ^2^ Mann–Whitney U test, ^3^ chi-square.

## Data Availability

The data underlying this article will be shared upon reasonable request to the corresponding author.
